# Proceedings of Ex-Confederate Surgeons

**Published:** 1898-07

**Authors:** 


### PROCEEDINGS OF EX-CONFEDERATE SURGEONS.

JULY 20, 1898.—The following surgeons and assistant surgeons organized temporarily, with Dr. J. McF. Gaston in the chair and Dr. V. G Hitt as Secretary: Drs. K. C. Divine, R. Y. Rudicil, W. H. Credile, V. G. Hitt, W. T. Goldsmith, J. P. Rosser, W. C. Smith, E. L. Connally, G. G. Roy, John R. Jones, J. W. Farrell, James E. H. Ware, John L. Branch, W. C. Smith, Edwin D. Norton and J. McF. Gaston, present.

There were enrolled forty-one surgeons and assistant surgeons before the Convention adjourned. Hospital Stewards were invited to join.

JULY 21.—Association was called to order at 9 A. M. by Dr. Gaston. As no permanent organization had been made heretofore, it was determined that this Association take the initiative, which was opposed by Dr. S. H. Stout, who thought that the surgeons should be organized through the various camps, with the Surgeon-General U. C. V. as directing head. This was overruled by all present. Dr. Stout retired and took no further part in the meetings. A nominating committee for permanent officers named Dr. J. B. Cowan, of Tullahoma, Tenn., as President; Dr. K. C. Divine, of Atlanta, Vice-President, and Dr. V. G. Hitt, Atlanta, Ga., as Secretary.

These gentlemen being duly elected, the Association indulged in discussions and relating various medical subjects and incidents as officers, both in hospital and field service.

The Presldent, Dr. J. B. Cowan, will appoint a surgeon from each State, and give due notice to collect all data of medical history.

The courtesies extended to the Convention by Dr. J. McF. Gaston by invitation to barbecue,* and by Dr. Todd, of Atlanta, to a reception at his home, were greatly appreciated.

The following was adopted by Committee and accepted by Association:

This Association shall be known as the Association of Surgeons of the Confederate Army and Navy. The object of said organization is to cultivate a friendly feeling among the members of the profession who served in the medical department of the army and navy of the Confederacy; also to collect, through its members, all matter pertaining to the medical service of the army and navy. All members of the medical profession who served as surgeons, assistant surgeons, contract physicians or hospital stewards are eligible to membership in said Association.

The following officers shall constitute the official list of said Association: President, Vice-President and Secretary, to be elected annually. The President shall appoint one from each State, who shall be authorized to collect all matter pertaining to the medical history of the Confederacy, the one so selected to be empowered to make such other appointments as, in their

*Given by Alumni of Southern Medical Collge.

discretion, may be deemed necessary to collect said material When collected said material to be forwarded to Surgeon-General U. C. V., Dr. C. H. Thebault, New Orleans, La., for compilation and preservation, or such other disposition as may be deemed proper by this Association. This Association shall meet annually at such time and place to be designated by the President.

J. J. KNOTT, Chairman.
R. Y. RUDICIL, Georgia.
JOHN R. JONES, Virginia.

Adopted unanimously.

It is expected that many papers of great interest will be presented at the next meeting in Charleston next year, and more perfect organization be made.

Association adjourned.

V. G. HITT.

---

### AN ARTICLE READ BEFORE THE MEETING OF THE EX-CONFEDERATE SURGEONS' ASSOCIATION.

During the latter part of the war I was assigned duty in charge of a railroad hospital in a small town where it was difficult to obtain medicines at almost any cost, and as I had my little hospital crowded nearly all the time both with employees of the road as well as wounded and sick soldiers afflicted with various diseases and all kinds of wounds and injuries, and also general practice in the surrounding country, it would naturally follow, that my mind was severely taxed in order to suppiy remedies and substitutes to meet the demands of such varied practice. I perused my dispensatory and called into requisition an old Botanic practice that had been handed down as a relic of the past, but from which I confess to have received valuable aid and very many useful hints in regard to the medical virtues of our native plants.

Having kept a record of all patients treated, and also remedies used in every case, from which I have been able to gather the following facts which show the general methods to which I resorted, and the various substitutes, at least the principal ones, upon which I mainly relied, I am not sure that in this paper I will be able either to offer anything new, entertaining or instructive to this intelligent and learned assemblage of surgeons, but yield to the request of those having this subject in charge.

I may say in the beginning that, of that large class of medicines so useful in surgery and so much in demand in war times, called antiseptics, many of them—I might say most of them–have been discovered and appropiated to surgical use since our late war; really I think the profession has advanced more and made more rapid strides in this particular line than in any other during the last one or two decades. In fact, I had but little else at my command except what we called the cold-water dressing for wounds and injuries; from closely watching and nursing, and I might say experimenting, I soon learned to improve on the plain old methods (as I thought, and still think). A decoction of red oak bark added to the water acted as disinfectant, and by its stimulating and astringent properties promoted the healing process considerably; also, a weak solution of bicarb. soda was especially beneficial in the suppurative stage. Often emollients were indicated; I used slippery elm and wahoo root bark, and as flax-seed, was scarce with me, at least; and solutions of common salt helped in many cases; and if painful poppy heads, nightshade (belladonna), stramonium would relieve pain.

A part of my railroad ran through a malarious section of the country and consequently I had considerable number of cases of intermittent fevers. And as I was frequently without quinine, I would give strong boneset tea, warm, until it produced free vomiting, then as a substitute for quinine I used during the intermissions plenty of *Cornus florida* (dog wood) bark tea or tea from the flowers, though the bark was preferable; also, red oak bark and common willow twig or bark tea; gave these teas freely during the intermisisons, and when fever rose I usually gave butterfly or pleurisy root tea, which would nearly always cut short the febrile stage.

Remittent or bilious fevers were treated pretty much the same way, except I invariably gave good doses of mandrake or mayapple tea in the beginning. This could be found in nearly all swamps and marshy places, etc. White ash root or prickly ash root were often given in these fevers to advantage, using always the butterfly root tea in febrile stage. Virginia snakeroot, yellow root or Sampson's snakeroot acted pretty nearly as well, though I preferred the other. Of course, if I had blue mass, calomel or other preparations of mercury I would begin treatment by giving a dose or two of that; but with none of these on hand, or quinine either, as was often the case, then the above course was pursued, which would not always act as quickly as quinine, but am sure quite as certainly if persevered in. Mayapple or peach-tree leaves, made in strong tea and drank warm, would act on the bowels as certain as senna; but, with children especially, when too much tea was not desirable, I often substituted beef's feet oil, hog's feet oil, or even lard heated with syrup.

In cases of pneumonia, pleurisy, catarrhal fevers, etc., I made local applications of mustard seed or leaves, stramonium leaves, hickory leaves, pepper, and warm applications, and gave alternately butterfly root and sanguiraria, and kept up to slightly nauseating continued from day to day (no need of anything else). These last two named remedies took the place of Dover's powder, quinine and all other diaphoretics, febrifuges, and arterial sedatives.

Phytolacca, or poke, was another favorite remedy; tincture when alcohol or whisky could be obtained, otherwise tea of root or berries. I used it in all cases of chronic rheumatism or neuralgia, enlarged glands, scrofula, syphilis, and all cases requiring alteratives, often combined with sarsaparilla root, sassafras, alder, and prickly ash.

Female complaints gave me some trouble, but I soon learned the use of black haw, squaw weed, partridge berry, etc. I had been taught in the old text-books that opiates in large doses would control some cases of threatened abortion, when the patient had not lost too much by hemorrhage; I found that the black haw root tea would absolutely stop this tendency, not only in cases where but little hemorrhage had taken place but in most cases where large quantities had passed, and would relieve the most severe case of dysmenorrhœa, especially when combined with squaw weed, partridge berry and red shank. Of late I often use these combined with cimicifuga.

In stomach and bowel diseases I found but little difficulty in finding plenty of substitutes for opiates, astringents and such like; in fact, I believe an All-wise Providence has especially provided the best antidotes in creation on the hills and dales, valleys and streams of our own Southland. In ordinary looseness of bowels or diarrheas an infusion of ordinary raspberry leaves or whortleberry leaves, both of which act finely on kidneys and bladder; where there is nausea of sick stomach, a hand full of peach-tree leaves steeped in water and drank will settle it; or, what is perhaps better, the kernels of two or three seeds, cracked and cold water drank off of it. If a stronger astringent is necessary, the inner bark of red oak, blackberry or dewberry root tea, or red shank root is one of the surest remedies. I was told by comrade Dr. Divine that he used the tea made from the dried leaves of this valuable plant, dried, and given in large quantities to the soldiers under his charge, with most decided benefit, during the prevalence of a most distressing epidemic of diarrhea. Agrimony tea and, as a last resort, the nut gall or ink ball, made into what I called from its color blackwash, made by squeezing the juice out, which is a scarlet red, put into a little copperas and it changes to a perfect black, and you not only have a splendid ink, but a destroyer of syphilitic sores, warts, corns, and old ulcers, and excrescences of nearly all kinds, much superior to lime and calomel. Blackwash weakened a little can be given in obstinate cases of bowel diseases and used as injection for gonorrhea, gleet, etc., and destroys ringworms and tetter. Ordinary silk wood root put in whisky and drank, giving at same time pills of rosin from pine trees with small pieces of blue vitriol in it, will cure obstinate cases of gonorrhea, and is a substitute for balsom, cubebs, etc. I raised lobelia from the seed; found it to be a reliable emetic, useful in cough medicine, croup and asthma. My mother was subject for many years to paroxysms of asthma, and always kept lobelia, which at nearly all times relieved her. I have seen her smoke life-everlasting and stramonium leaves with marked effect. We, of course, used spirits of turpentine as an adjunct in all cases when indicated, which is the case in very many diseases and in many cases a positive curative agent. Onions and garlic were useful for poultices in nearly all glandular enlargements, as is also phytolacca root. Celery, pepper, parsley, sage, thyme, rhue, and several varieties of garden products were brought into requisition, mostly used for teas for children and for female disorders. We paid special attention to dietetic preparations, as we had to manufacture most of them from potatoes, wheat, rice, etc. White sumac, red elm, prickly ash and poke will, in connection with my blackwash, cure recent cases of syphilis. It will also cure many cases of chronic rheumatism. Peachtree leaves and Sampson's snakeroot will relieve most cases of incipient dyspepsia. Gargles made of poplar bark with sage and honey will cure most cases of sore throat, tonsilitis, etc.

For infants, calamus, catnip and soot tea is better than soothing syrups with opiates. As a substitute for flaxseed for poultices, wahoo, slippery elm, or, what is still better, the thick pulpy inside mass of cactus leaves; put in fire, burn off stickers and it will be soft and just suited for emollient poultices. —W. C. Smith, Atlanta Ga.

---

ATLANTA, August 4, 1898.

I desire to state that the publishing of my name as Professor of Physiology in the Dental Department of the Atlanta College of Physicians and Surgeons is not only utterly unwarranted, but has been done in the face of my having declined positively to accept said position.

JOHN C. OLMSTED, M.D.

